# Nonvolatile Chemical Constituents from the Leaves of *Ligusticopsis wallichiana* (DC.) Pimenov & Kljuykov and Their Free Radical-Scavenging Activity

**DOI:** 10.1155/2018/1794650

**Published:** 2018-02-01

**Authors:** Hari Prasad Devkota, Bibek Adhikari, Takashi Watanabe, Shoji Yahara

**Affiliations:** ^1^Graduate School of Pharmaceutical Sciences, Kumamoto University, 5-1 Oe-honmachi, Chuo ku, Kumamoto 862-0973, Japan; ^2^Program for Leading Graduate Schools, Health Life Science: Interdisciplinary and Glocal Oriented (HIGO) Program, Kumamoto University, Kumamoto, Japan

## Abstract

Different plant parts of *Ligusticopsis wallichiana* (family: Apiaceae) are widely used as traditional medicines. Although many volatile constituents are already identified from the leaves of *L. wallichiana*, there is no detailed report on the nonvolatile constituents. In the present study, we aimed to isolate and identify the major chemical constituents from the leaves. Bhutkesoside A (**1**), falcarindiol (**2**), ferulic acid (**3**), cnidioside A (**4**), quercetin 3-*O*-*β*-D-glucopyranoside (**5**), rutin (**6**), 4′-*O*-methylquercetin 3-*O*-*β*-D-glucopyranoside (**7**), scopoletin (**8**), umbelliferone (**9**), eugenol 4-*O*-*β*-D-glucopyranoside (**10**) and pumilaside A (**11**) were isolated from the 70% MeOH extract. The structures of isolated compounds were elucidated on the basis of ^1^H- and ^13^C-NMR spectroscopic data. Compounds **4–11** are reported for the first time from *L. wallichiana*. Compounds **5** and **6** showed potent free radical-scavenging activity.

## 1. Introduction

*Ligusticopsis wallichiana* (DC.) Pimenov & Kljuykov (Syns. *Selinum wallichianum* (DC.) Raizada & H. O. Saxena, *Selinum tenuifolium* Wall. ex C. B. Clarke) is a perennial aromatic herb belonging to family Apiaceae. It is widely distributed in the Himalayan region of Nepal, India, Pakistan, Bhutan, and China between 2700 and 4800 m [1, 2]. In Nepal, it is locally known as “*Bhutkesh*” and the root decoction is used to treat body pain, fever, cough, and cold [1]. Flowers and leaves in the form of infusions are used to treat stomachache, and they are also applied locally for healing cuts and wounds [3]. In India, the root decoction is used for the treatment of diarrhea, stomachache, and vomiting. The flowers and stems are used for stimulant and carminative properties [2]. Previous studies on *L. wallichiana* were mainly focused on the volatile constituents of the different plant parts [2–6], but there is no detailed report on the nonvolatile constituents from the leaves. Recently, we reported two novel compounds, bhutkesoside A (**1**) and bhutkesoside B and ten known compounds from the roots of the same plant [7]. On continuation, in this paper, we report the detailed isolation and spectroscopic identification of major chemical constituents from the leaves of *L. wallichiana* and 1,1-diphenyl-2-picrylhydrazyl (DPPH) free radical-scavenging activity of isolated compounds.

## 2. Experiment

### 2.1. General Experimental Procedures

^1^H-, ^13^C-, and 2D-NMR spectra were measured on a JEOL *α* − 500 (^1^H-NMR: 500 MHz and ^13^C-NMR: 125 MHz). Chemical shifts are given in ppm with reference to tetramethyl silane (TMS). Mass spectra were recorded on JEOL JMS-700 MStation. Absorbance was recorded on Immuno-MiniNJ-2300 Microtiter Plate Reader, Biotech Pvt., Ltd. (Tokyo, Japan). Column chromatography was carried out with MCI gel CHP20P (75∼150 *μ*m, Mitsubishi Chemical Industries Co., Ltd., Tokyo, Japan), Sephadex LH-20 (Amersham Pharmacia Biotech, Tokyo, Japan), Chromatorex ODS (30∼50 *μ*m, Fuji Silysia Chemical Co., Ltd., Aichi, Japan), and silica gel 60 (0.040–0.063 mm, Merck KGaA, Darmstadt, Germany). TLC was performed on a precoated silica gel 60 F_254_ (aluminum sheet, Merck KGaA, Darmstadt, Germany).

### 2.2. Chemicals

1,1-Diphenyl-2-picrylhydrazyl (DPPH) and Trolox were purchased from Wako Pure Chemicals, Osaka, Japan, and MES buffer was purchased from Dojindo Chemical Research, Kumamoto, Japan.

### 2.3. Plant Material

The fresh leaves of *L. wallichiana* were collected from Kurikharkha, Dolkha, Nepal in August 2013. The plant specimen was identified by Mr. Kuber Jung Malla, Senior Scientific Officer, Department of Plant Resources, Nepal. The voucher specimen (Voucher Number: KUNP20130809-015) was deposited at the Museum of Graduate School of Pharmaceutical Sciences, Kumamoto University, Kumamoto, Japan.

### 2.4. Extraction and Isolation

The shade dried leaves (540.0 g) were macerated three times (48 hours for each time) with 70% MeOH (8 L) at room temperature with frequent stirring. The extracts were then combined and evaporated under reduced pressure to give 131.0 g of semisolid extract. A part of the extract (119.0 g) was subjected to MCI gel CHP20P CC and eluted successively with water, 40%∼100% MeOH to afford fifteen fractions (1∼15). Fraction 6 (2.0 g, 40% MeOH eluate) was subjected successively to Sephadex LH-20 CC (50% MeOH), ODS CC (10% MeOH), and silica gel column (CHCl_3_  :  MeOH  :  H_2_O = 8  :  2  :  0.1) to obtain compound **4** (7.6 mg) and **11** (21.4 mg). Fraction 7 (2.6 g, 40% MeOH eluate) was applied over Sephadex LH-20 CC (50% MeOH) and ODS CC (20∼25% MeOH) to give compounds **5** (34.4 mg) and **6** (127.0 mg). Fraction 9 (2.0 g, 70% MeOH eluate) was applied over Sephadex LH-20 CC (50∼100% MeOH) to give ten subfractions (9–1∼10). Subfraction 9–2 (927.0 mg) was applied over Sephadex LH-20 CC (40% MeOH) silica gel column (CHCl_3_  :  MeOH = 10  :  1), and ODS CC (20% MeOH) to give compounds **1** (13.2 mg) and **10** (20.1 mg). Subfraction 9–4 (321.6 mg) was subjected on silica gel column (hexane  :  EtOAc = 3  :  2) to obtain compounds **8** (29.2 mg) and **9** (15.6 mg). Subfraction 9–5 was obtained as compound **3** (77.3 mg). Fraction 11 (875 mg, 70% MeOH eluate) was applied over Sephadex LH 20 and ODS CC (40–42% MeOH) to give compound **7** (6.0 mg). Fraction 13 (1.89 g, MeOH eluate) was applied over silica gel column (hexane  :  EtOAc = 3  :  1) to give compound **2** (375 mg).

### 2.5. Measurement of Free Radical-Scavenging Activity

The DPPH radical-scavenging activity of the isolated compounds was measured by the method as described by Li and Seeram [8] with slight modifications. Briefly, 50 *μ*L of 200 mM MES (2-(*N*-morpholino) ethanesulphonic acid) buffer (pH 6.0), 100 *μ*L of samples at different concentrations (in DMSO  :  ethanol = 1  :  1) and 50 *μ*L of 800 mM DPPH in ethanol solution were mixed in a 96-well plate and kept in dark at room temperature for 20 minutes. The radical-scavenging activity was measured at 510 nm with UV spectrophotometer using the following formula: radical-scavenging activity (%) = 100 × (*A* − *B*)/*A*. where *A* is the control absorbance of DPPH radicals without samples and *B* is the absorbance after reacting with samples. Trolox was used as the positive control. From these data, curve was plotted and effective concentration (EC_50_) value was calculated which is defined as the concentration (*μ*M) of the compound required for 50% reduction of the DPPH radical absorbance.

## 3. Results and Discussion

**Figure 1:**
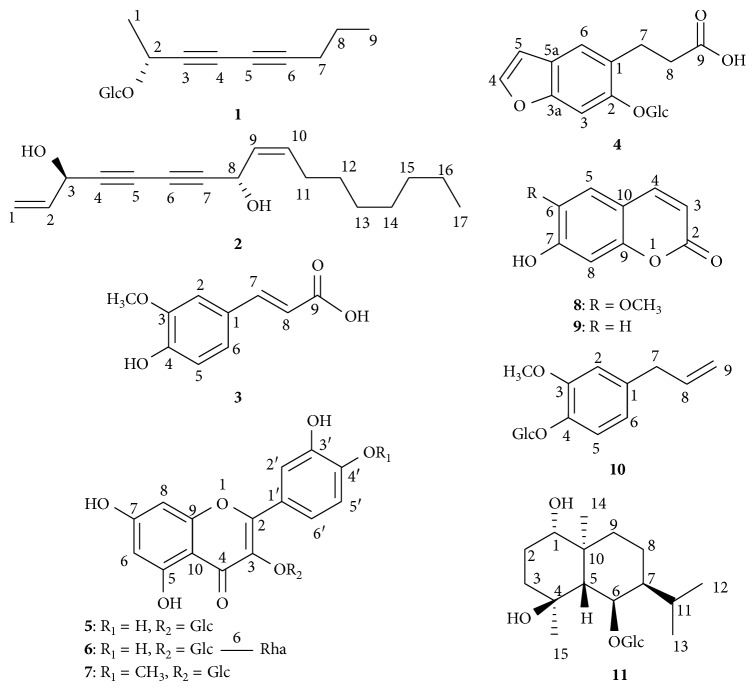
Structures of isolated compounds.

The shade dried leaves of *L. wallichiana* were extracted with 70% MeOH, and the extract was then subjected to repeated column chromatography (CC) on MCI gel CHP20P, Sephadex LH20, ODS, and silica gel column to obtain 11 compounds (**1–11**). Structures of these compounds were determined on the basis of ^1^H- and ^13^C-NMR spectroscopic data and comparison with reference values (Figure 1).

Compound **1**, a pale yellow oil, [*α*]_*D*_^27^ −117° (*c* = 0.35, MeOH), was identified as 2(*R*)-hydroxy-3,5-nonadiyn-2-*Ο*-*β*-D-glucopyranoside named as bhutkesoside A, which was a new diacetylene glucoside isolated from the roots of *L. wallichiana* in our previous study [7]. The detailed ^1^H- and ^13^C-NMR data for compound **1** are given in Table 1.

Compound **2** was obtained as yellowish orange oil, [*α*]_*D*_^20^ +99.9° (*c* = 0.88, MeOH). The ^1^H-NMR spectrum (Table 2) showed three proton signals at *δ*_H_ 5.94 (1H, ddd, *J* = 5.2, 10.4, 17.2 Hz), 5.47 (1H, dt, *J* = 1.5, 17.2 Hz), and 5.25 (1H, dt, *J* = 1.5, 10.4 Hz) assignable to terminal vinyl protons. A set of olefenic protons at *δ*_H_ 5.51 (1H, dd, *J* = 8.5, 10.6 Hz) and 5.61 (1H, ddd, *J* = 1.2, 7.3, 10.6 Hz) were also present and their coupling constant of 10.6 Hz suggested the *cis* configuration. Two protons attached to oxygen bearing carbon were present at *δ*_H_ 5.20 (1H, brd, *J* = 8.5 Hz) and *δ*_H_ 4.93 (1H, d, *J* = 5.2 Hz). Two methylene proton at *δ*_H_ 2.10 to 1.27 ppm and a methyl signal at *δ*_H_ 0.88 (3H, t, *J* = 7.0 Hz) were also observed. The ^13^C-NMR spectra (Table 2) showed signals equivalent to total seventeen carbons. The natures of these carbons were determined by DEPT spectra. Among these carbon signals, four quaternary carbon signals at *δ*_C_ 78.2 (C), 70.3 (C), 79.8 (C), and 68.7 (C) were assignable to a disubstituted acetylene moiety. Two oxygen-bearing carbons at *δ*_C_ 63.4 (CH) and 58.5 (CH) and a methyl group at *δ*_C_ 14.0 ppm were also observed. On the basis of these data and comparison with literature values, compound **2** was identified as falcarindiol [9].

Compound **3** was obtained as white needles. The ^1^H-NMR spectrum (Table 3) showed three proton signals in the aromatic region at *δ*_H_ 7.27 (1H, *J* = d, 2.1 Hz), 7.08 (1H, dd, *J* = 2.1, 8.2 Hz), and 6.80 (1H, *J* = d, 8.2 Hz) assignable to a 1,3,4-trisubstituted aromatic ring. Two signals at *δ*_H_ 7.51 (1H, d, *J* = 15.9 Hz) and 6.36 (1H, d, *J* = 15.9 Hz) suggested presence of *trans* olefenic protons. A methoxy signal at *δ*_H_ 3.82 (3H, s) attached to aromatic ring was also observed. The ^13^C-NMR (Table 3) showed six carbon signals at *δ*_C_ 149.1 (C), 147.9 (C), 125.8 (C), 122.8 (CH), 115.6 (CH), and 111.2 (CH), which confirmed a 1,3,4-trisubstituted aromatic ring and signals at *δ*_C_ 144.6 (CH) and 115.5 (CH) confirmed a *trans* olefenic moiety. Further quaternary carbon at *δ*_C_ 168.1 (C) for a carbonyl carbon and methoxy signal at *δ*_C_ 55.7 (OCH_3_) were also observed. On the basis of these data and comparison with literature values, compound **3** was identified as ferulic acid [10].

Compound **4** was obtained as white amorphous powder, [*α*]_*D*_^27^ −33.4° (*c* = 0.45, pyridine). The ^1^H-NMR spectrum (Table 4) of compound **4** showed four aromatic or olefinic protons at *δ*_H_ 7.82 (1H, d, *J* = 2.2 Hz), 7.37 (1H, s), 7.32 (1H, s), and 6.81 (1H, d, 2.2 Hz). Seven proton signals attached to oxygen bearing carbons assignable to a sugar moiety were present at *δ*_H_ 4.82 (1H, d, *J* = 7.3 Hz), 3.72 (1H, dd, *J* = 5.01, 11.8 Hz), 3.26–3.30 (2H, br, m), 3.16 (2H, t, *J* = 9.1 Hz), and 3.75 (1H, dd, *J* = 1.8, 11.8 Hz). The proton signals at *δ*_H_ 4.82 (1H, d, *J* = 7.3 Hz) was assignable to the anomeric proton for a sugar moiety. Further two methylene protons signals coupled each other at *δ*_H_ 2.87 (2H, t, *J* = 7.6 Hz) and 2.40 (2H, t, *J* = 7.6 Hz) were observed. The ^13^C-NMR spectra (Table 4) showed signals equivalent to total eighteen carbons and among them eight aromatic carbons at *δ*_C_ 153.5 (C), 153.4 (C), 144.9 (CH), 126.6 (C), 120.8 (C), 120.6 (CH), 106.2 (CH), and 98.3 (CH) revealed a benzofuran moiety. Six carbon signals at *δ*_C_ 101.5 (CH), 73.3 (CH), 77.0 (CH), 69.9 (CH), 76.6 (CH), and 60.8 (CH_2_) were assignable to a *β*-glucopyranosyl moiety which was also supported by the coupling constant of anomeric proton (*J* = 7.3 Hz). Three carbon signals *δ*_C_ 174.8 (C), 35.3 (CH_2_), and 26.1 (CH_2_) revealed a propanoic acid derivative. On the basis of these data and comparison with literature values, compound **4** was identified as cnidioside A [11].

Compound **5** was obtained as pale yellow crystalline powder, [*α*]_*D*_^23^ −31.9° (*c* = 0.99, pyridine). The ^1^H-NMR spectrum (Table 5) showed five proton signals at *δ*_H_ 7.59 (1H, d, *J* = 2.2 Hz), 7.58 (1H, brd, *J* = 8.4 Hz), 6.85 (1H, d, *J* = 8.4 Hz), 6.41 (1H, d, *J* = 1.8 Hz), and 6.21 (1H, d, *J* = 1.8 Hz) assignable to proton signals of quercetin. Seven proton signals attached to oxygen bearing carbon were present at *δ*_H_ 5.47 (1H, d, *J* = 7.0 Hz), 3.10 (1H, m), 3.25 (2 H, m), 3.10 (1H, m), 3.59 (1H, d, *J* = 11.3 Hz), and 3.33 (1H, d *J* = 11.3 Hz) were present. Among them proton at *δ*_H_ 5.47 (1H, d, *J* = 7.0 Hz) was assignable to anomeric proton of the sugar moiety. The ^13^C-NMR spectra of (Table 5) showed signals equivalent to total twenty-one carbons, in which 15 carbon signals at *δ*_C_ 177.5 (C), 164.3 (C), 161.2 (C), 156.3 (C), 156.2 (C), 144.8 (C), 148.5 (C), 133.4 (C), 121.6 121.2 (C), 115.2 (CH), 116.2 (CH), 104.0 (C), and 98.7 (CH), were assignable to a 3-*O*-substituted quercetin. The remaining six signals at *δ*_C_ 100.9 (CH), 77.5 (CH), 76.5 (CH), 74.1 (CH), 69.9 (CH), and 61.0 (CH_2_) for a monosaccharide revealed the *β*-glucopyranosyl moiety which was supported by the coupling constant (*J* = 7.0 Hz) of anomeric proton. On the basis of these data and comparison with literature values, compound **5** was identified as quercetin 3-*O*-*β*-D-glucopyranoside [12].

Compound **6** was obtained as pale yellow crystalline powder, [*α*]_*D*_^23^ −35.5° (*c* = 0.37, pyridine). The ^1^H-NMR spectrum (Table 5) of compound **6** was similar to that of compound **5** except some additional signals of sugar moiety. Proton signal for anomeric proton at 5.29 (1H, brs) and a methyl group at *δ*_H_ 1.00 (3H, d, *J* = 6.1 Hz) suggested the presence of rhamnopyranosyl moiety. The ^13^C-NMR spectra (Table 5) showed signals equivalent to total twenty-seven carbons. Similar to compound **5**, fifteen carbon signals were assignable to a 3-*O*-substituted quercetin moiety. Among the remaining 12 signals, six signals at *δ*_C_ 101.2 (CH), 76.4 (CH), 75.9 (CH), 74.1 (CH), 70.6 (CH), and 68.2 (CH_2_) were assignable to a *β*-glucopyranosyl moiety and other six carbons signals at *δ*_C_ 100.7 (CH), 70.4 (CH), 70.0 (CH), 71.8 (CH), 67.0 (CH), and 17.7 (CH_3_) were assignable to a *α*-rhamnopyranosyl moiety. The downfield shift of C-6 of glucopyranosyl moiety at 68.2 ppm suggested Rha-1→Glc-6 linkage. On the basis of these data and comparison with literature values, compound **6** was identified as rutin [12].

Compound **7** was obtained as yellow powder, [*α*]_*D*_^27^ −14.7° (*c* = 0.60, pyridine). The ^1^H-NMR spectrum (Table 5) showed signals similar to compound **5** except an additional signal for methoxy group at *δ*_H_ 3.85 (3H, s). The ^13^C-NMR spectra of compound **7** (Table 5) showed signals equivalent to total twenty-two carbons, in which 15 carbon signals at *δ*_C_ 177.5 (C), 164.3 (C), 161.2 (C), 156.3 (C), 156.2 (C), 144.8 (C), 148.5 (C), 133.4 (C), 121.6 121.2 (C), 115.2 (CH), 116.2 (CH), 104.0 (C), and 98.7 (CH), were assignable to a 3-*O*-substituted quercetin. The remaining six signals at *δ*_C_ 101.5 (CH), 72.7 (CH), 70.8 (CH), 68.2 (CH), and 63.7 (CH_2_) confirmed the presence of a *β*-glucopyranosyl which was also supported by the coupling constant (*J* = 7.9 Hz) of the anomeric proton. Signal at *δ*_C_ 55.6 was assigned to a methoxy group. In differential NOE experiment, irradiation of the methoxy signal at *δ*_H_ 3.85 (3H, s) increased the intensity of proton signal assignable to C-5′ at *δ*_H_ 7.04 (d, 8.4 Hz) while no effect was seen in the protons at C-2′ at *δ*_H_ 7.56, 1H (d, 2.2 Hz), which suggested that the methoxy group was attached at C-4′ position in B-ring of quercetin. On the basis of these data and comparison with literature values, compound **7** was identified as 4′-*O*-methylquercetin 3-*O*-*β*-D-glucopyranoside [13].

Compound **8** was obtained as white crystals. The TLC spot for compound **8** showed blue colour under UV (365 nm), suggesting a coumarin derivative. The ^1^H-NMR spectrum (Table 6) showed four proton signals in aromatic or olefenic region at *δ*_H_ 7.84 (1H d, *J* = 9.5 Hz), 7.10 (1H, s), 6.76 (1H, s), and 6.20 (1H, d, *J* = 9.5 Hz). Further a proton singlet at *δ*_H_ 3.90 (3H, s) suggested a methoxy group. The ^13^C-NMR spectra (Table 6) showed signals equivalent to total 10 carbons and among them, 9 carbon signals at *δ*_C_ 164.1 (C), 152.9 (C), 151.5 (C), 147.1 (C), 146.1 (CH), 112.6 (C), 112.7 (CH), 110.0 (CH), and 104.0 (CH) were assignable to a 6,7-dihydroxycoumarin derivative. Moreover, signal at *δ*_C_ 56.8 was assigned to a methoxy group. In differential NOE experiment, irradiation of a proton signal at 7.10 *δ*_H_ (1H, s) assignable to proton attached to C-5 position of coumarin increased the intensity of methoxy signal at *δ*_H_ 3.90 (3H, s) as well as proton assignable to C-4 position at 7.84 (1H d, *J* = 9.5 Hz), which suggested that the methoxy group was attached to C-6 position. On the basis of these data and comparison with literature values, compound **8** was identified as scopoletin [14].

Compound **9** was obtained as white crystals. The TLC spot for compound **9** also showed blue colour under UV (365 nm), suggesting a coumarin derivative. The ^1^H-NMR spectrum (Table 6) showed five protons in the aromatic region two proton signals coupled each other at *δ*_H_ 6.18 (1H, d, *J* = 9.5 Hz) and 7.84 (1H d, *J* = 9.5 Hz) and three proton signals at *δ*_H_ 7.45 (1H, d, *J* = 8.5 Hz), 6.77 (1H, dd, *J* = 2.4, 8.5 Hz), and 6.70 (1H, d, *J* = 2.4 Hz), suggesting that the compound **9** was a 7-hydroxycoumarin. The ^13^C-NMR spectra (Table 6) showed signals equivalent to total 9 carbons at *δ*_C_ 163.7 (C), 163.1 (C), 157.2 (C), 146.0 (CH), 130.7 (CH), 114.5 (CH), 111.6 (C), 112.3 (CH), and 103.4 (CH). These carbon signals were superimposable with that of umbelliefone [14].

Compound **10** was obtained as white amorphous powder, [*α*]_*D*_^27^ −35.5° (*c* = 0.92, pyridine). The ^1^H-NMR spectrum (Table 7) showed three proton signals in the aromatic region at *δ*_H_ 6.81 (1H, d, *J* = 1.8 Hz), 7.07 (1H, d, *J* = 8.2 Hz), and 6.71 (1H, dd, *J* = 8.2, 1.8 Hz), which were assignable to a 1,3,4-substituted aromatic ring. Further three proton signals due to terminal alkene at *δ*_H_ 5.94 (1H, ddt, *J* = 17.0, 10.2, 6.7 Hz), 5.05 (1H, dd, *J* = 17.0, 1.8 Hz), and 5.01 (1H, dd, *J* = 10.2, 1.8 Hz) coupled with two proton signals at *δ*_H_ 3.31 (2H, d, *J* = 6.7 Hz) were observed, which suggested the presence of allyl moiety. A singlet at *δ*_H_ 3.83 (3H, s) suggested the presence of a methoxy group. Further seven protons assignable to a sugar moiety were present at *δ*_H_ 4.83 (1H, d, *J* = 7.3 Hz), 3.48 (1H, dd, *J* = 7.3, 9.1 Hz), 3.37–3.39 (2H, m), 3.45 (1H, dd, *J* = 9.1, 9.8 Hz), 3.68 (1H, dd, *J* = 12.2, 5.4 Hz), and 3.86 (1H, dd, *J* = 12.2, 1.5 Hz). The ^13^C-NMR spectra (Table 7) showed signals equivalents to total 16 carbons, in which 9 carbon signals at *δ*_C_ 150.8 (C), 146.4 (C), 139.0 (CH), 136.5 (C), 122.1 (CH), 118.3 (CH), 115.1 (CH_2_), 114.2 (CH), and 40.7 (CH_2_), were assignable to a 1,3,4-trisubstituted aromatic ring with an allyl moiety. Other six signals *δ*_C_ 103.1 (CH), 78.2 (CH), 77.9 (CH), 74.9 (CH), 71.4 (CH), and 62.5 (CH_2_) were assignable to *β*-glucopyranosyl moiety as in the case of previous compounds. The carbon signal at *δ*_C_ 56.8 was assigned to a methoxy group. In differential NOE experiment, irradiation of the methoxy signal at *δ*_H_ 3.83 (3H, s) enhanced the intensity of proton at *δ*_H_ 6.79 (1H, d, *J* = 1.8 Hz) assignable to C-2 of the aromatic ring, which suggested that the methoxy group was present at C-3 position, and glucopyranosyl moiety was attached to C-4. On the basis of these data and comparison with literature values, compound **10** was identified as eugenol 4-*O*-*β*-D-glucopyranoside [15].

Compound **11** was obtained as colourless gum, [*α*]_*D*_^27^ −25.9°. Its molecular formula was determined to be C_21_H_38_O_8_ on the basis of a HR-FAB-MS peak of [M + Na]^+^ at 441.2482 (calculated for C_21_H_38_O_8_Na, 441.2464). ^1^H-NMR spectrum of compound **11** (Table 8) showed several proton signals from 1 to 2 ppm, clear signals for two methyl doublets were present at *δ*_H_ 0.90 (3H, d, *J* = 6.7 Hz) and 1.03 (3H, d, *J* = 6.7 Hz), two methyl singlets were present at *δ*_H_ 0.83 (3H, s) and 1.23 (3H, s). Remaining ten proton signal equivalents were present from *δ*_H_ 2.11 to *δ*_H_ 4.48 ppm. The ^13^C-NMR spectra (Table 8) showed total 21 carbon signals and among them, six signals at *δ*_C_ 98.3 (CH), 77.0 (CH), 76.9 (CH), 74.3 (CH), 70.4 (CH), and 61.4 (CH_2_) were assignable to a glucopyranosyl moiety. The rest fifteen signals at *δ*_C_ 77.9 (CH), 76.8 (CH), 71.3 (C), 49.9 (CH), 40.3 (CH), 39.9 (CH_2_), 35.5 (CH_2_), 27.9 (CH_2_), 24.6 (CH), 23.6 (CH_3_), 23.1 (CH_3_), 22.6 (CH_3_), 22.4 (CH_2_), and 13.8 (CH_3_) can be assignable to a eudesmane-type sesquiterpenoid moiety. On the basis of these data and comparison with literature values, compound **11** was identified as pumilaside A [16].

Among these eleven compounds isolated from the leaves of *L. wallichina* in this study, a diacetylene glucoside, bhutkesoside A (**1**); a polyacetylene derivative, falcarindiol (**2**); and a phenylpropanoid derivative, ferulic acid (**3**) were also isolated from the roots of same plant, which were reported in a previous paper [7]. All other compounds (**4–11**) were isolated for the first time from this plant which included a benzofuran derivative, cnidioside A (**4**); three flavonoid derivatives, quercetin 3-*O*-*β*-D-glucopyranoside (**5**), rutin (**6**), and 4′-*O*-methylquercetin 3-*O*-*β*-D-glucopyranoside (**7**); two coumarin derivatives, scopoletin (**8**) and umbelliferone (**9**); a phenylpropene derivative, eugenol 4-*O*-*β*-D-glucopyranoside (**10**), and a eudesmane sesquiterpene glucoside, pumilaside A (**11**). It was the first study on the isolation and identification of nonvolatile compounds from the leaves of *L. wallichiana*. Regarding coumarin derivatives, three furocoumarins such as bergapten, heraclenin, and heraclenol were isolated from the roots of *L. wallichiana* [17]. This is the first report on the presence of flavonoids in *L. wallichiana* and presence of coumarin derivatives in the leaves.

All these isolated compounds were evaluated for their DPPH free radical-scavenging activity. Among them, only two flavonoids, rutin (**6**) (EC_50_ 52.4 *μ*M) and quercetin-3-*O*-*β*-D-glucopyranoside (**5**) (EC_50_ 54.5 *μ*M) showed potent free radical-scavenging activity as compared to positive control, Trolox (EC_50_ 96.1 *μ*M). Flavonoids with unsubstituted hydroxyl groups in C_3′_ and C_4′_ position (**5** and **6**) showed potent activity; however, a compound with C-4′ methoxy substitution, 4′-*O*-methylquercetin 3-*O*-*β*-D-glucopyranoside (**7**), did not show any activity in the free radical-scavenging assay. These results were similar to previous studies on the free radical-scavenging activities of flavonoids [18, 19].

In conclusion, eleven nonvolatile compounds belonging to different chemical classes were isolated and identified for the first time from the leaves of *L. wallichiana*. Some of the isolated compounds also showed potent free radical-scavenging activity. Further studies should focus on the detailed biological activities of extracts and isolated compounds to provide the scientific evidence for their traditional uses.

## Figures and Tables

**Table 1:** ^1^H- and^13^C-NMR spectroscopic data of compound **1** in CD_3_OD.

Carbon number	*δ*_H,_ mult. (*J* in Hz)	*δ*_C_
1	1.42, d (6.7)	22.3, CH_3_
2	4.80, brs	64.2, CH
3	—	75.7, C
4	—	71.3, C
5	—	65.4, C
6	—	82.0, C
7	2.27, t (7.3)	21.7, CH_2_
8	1.55, dq (7.3)	22.8, CH_2_
9	1.00, t (7.3)	13.7, CH_3_
Glc-1	4.55, d (7.6)	101.3, CH
Glc-2	3.17, dd (7.6, 9.2)	74.9, CH
Glc-3	3.38, t (9.2)	78.0, CH
Glc-4	3.27, m	71.7, CH
Glc-5	3.27, m	78.1, CH
Glc-6	3.80, dd (11.5, 1.5)	62.8, CH_2_
	3.64, dd (11.5, 5.4)	

**Table 2:** ^1^H- and^13^C-NMR spectroscopic data of compound **2** in CDCl_3_.

Carbon number	*δ*_H,_ mult. (*J* in Hz)	*δ*_C_
1	5.47, dt (17.2, 1.5)	117.3, CH_2_
	5.25, dt (10.4, 1.5)	
2	5.94, ddd (5.2, 10.4, 17.2)	135.7, CH
3	4.93, brd (5.2)	63.4, CH
4	—	78.2, C
5	—	70.3, C
6	—	68.7, C
7	—	79.8, C
8	5.20, brd (8.5)	58.5, CH
8		
9	5.51, brdd (8.5, 10.6)	127.6, CH
10	5.61, ddd (1.2, 7.3, 10.6)	134.6, CH
11	2.10, dq (1.2, 7.3)	31.7, CH_2_
12	1.38, t like (7.3)	29.2, CH_2_
13	1.27–1.29, m	29.2, CH_2_
14	1.27–1.29, m	29.2, CH_2_
15	1.27–1.29, m	27.2, CH_2_
16	1.27–1.29, m	22.1, CH_2_
17	0.88, t (7.0)	14.0, CH_3_

**Table 3:** ^1^H- and^13^C-NMR spectroscopic data of compound **3** in DMSO-*d*_6_.

Carbon number	*δ*_H,_ mult. (*J* in Hz)	*δ*_C_
1	—	125.8, C
2	7.27, d (2.1)	111.2, CH
3	—	149.1, C
4	—	147.9, C
5	6.80, d (8.5)	115.6, CH^a^
6	7.08, dd (2.1, 8.2)	122.8, CH
7	7.51, d (15.9)	144.6, CH
8	6.36, d (15.9)	115.5, CH^a^
9	—	168.1, C
OCH_3_	3.82, s	55.7, OCH_3_

^a^Assignments with the same superscript may be interchanged in the same column.

**Table 4:** ^1^H- and^13^C-NMR spectroscopic data of compound **4** in CD_3_OD.

Carbon number	*δ*_H,_ mult. (*J* in Hz)	*δ*_C_
1	—	126.6, C^a^
2	—	153.5, C^b^
3	7.37, s	98.3, CH
3a	—	153.4, C^b^
4	7.82, d (2.2)	144.9, CH
5	6.81, d (2.2)	106.2, CH
5a	—	120.8, C^a^
6	7.32, s	120.6, CH
7	2.87, t (7.6)	26.1, CH_2_
8	2.40, t (7.6)	35.3, CH_2_
9	—	174.8, C
Glc-1	4.82, d (7.3)	101.5, CH
Glc-2	3.26–3.30, m	73.3, CH
Glc-3	3.16, t (9.1)	77.0, CH^c^
Glc-4	3.16, t (9.1)	69.9, CH
Glc-5	3.26–3.30, m	76.6, CH^c^
Glc-6	3.72, dd (5.0, 11.8)	60.8, CH_2_
	3.75, dd (1.8, 11.8)	

^a,b,c^Assignments with the same superscripts may be interchanged in the same column.

**Table 5:** ^1^H- and^13^C-NMR spectroscopic data of compounds **5**, **6**, and **7** in DMSO-*d*_6_.

Carbon number	**5**		**6**		**7**	
	*δ*_H,_ mult. (*J* in Hz)	*δ*_C_	*δ*_H,_ mult. (*J* in Hz)	*δ*_C_	*δ*_H,_ mult. (*J* in Hz)	*δ*_C_
2	—	156.3, C^a^	—	156.4, C^b^	—	156.2, C^a^
3	—	133.4, C	—	133.3, C	—	133.5, C
4	—	177.5, C	—	177.4, C	—	177.4, C
5	—	161.2, C^c^	—	161.2, C^d^	—	161.2, C^c^
6	6.21, d (1.8)	98.7, CH	6.19, d (2.2)	100.7, CH^e^	6.20, d (1.8)	98.7, CH
7	—	164.3, C^c^	—	164.0, C^d^	—	164.1, C^c^
8	6.41, d (1.8)	93.5, CH	6.38, d (2.2)	93.6, CH	6.41, d (1.8)	93.6, CH
9	—	156.2,C^a^	—	156.6, C^b^	—	155.7, C^a^
10	—	104.0, C	—	104.0, C	—	103.9, C
1′	—	121.2, C	—	121.2, C	—	122.6, C
2′	7.59, d (2.2)	115.2, CH^f^	7.52, d (2.2)	115.2, CH^f^	7.56, d (2.2)	115.7, CH^f^
3′	—	144.8, C	—	144.7, C	—	145.8, C
4′	—	148.5, C	—	148.4, C	—	149.9, C
5′	6.85, d (8.4)	116.2, CH^f^	6.81, d (8.4)	116.3, CH^f^	7.04, d (8.4)	114.8, CH^f^
6′	7.58, brd (8.4)	121.6, CH	7.54, brd (8.4)	121.6, CH	7.70, brd (8.4)	121.4, CH
OCH_3_	—	—	—	—	3.85, s	55.6, OCH_3_
Glc-1	5.47, d (7.0)	100.9, CH	5.34, d (7.3)	101.2, CH^e^	5.48, d (7.0)	100.8, CH
Glc-2	3.10, m	74.1, CH	3.22–3.42, m	74.1, CH	3.09, m	74.0, CH
Glc-3	3.24–3.25, m	77.5, CH^e^	3.22–3.42, m	76.4, CH^c^	3.17–3.24, m	77.5, CH^e^
Glc-4	3.10, m	69.9, CH	3.07, dd (9.5, 9.2)	70.6, CH^a^	3.09, m	69.8, CH
Glc-5	3.24–3.25, m	76.5, CH^e^	3.22–3.42, m	75.9, CH^c^	3.17–3.24, m	76.4, CH^e^
Glc-6	3.59, brd (11.3)	61.0, CH_2_	3.67, brd (10.2)	67.0, CH_2_	3.57, brd (11.3)	60.9, CH_2_
	3.33, brd (11.3)		3.70, brd (10.9)		3.60, m	
Rha-1	—	—	5.29, brs	100.7, CH^e^	—	—
Rha-2	—	—	3.22–3.42, m	70.4, CH^a^	—	—
Rha-3	—	—	3.22–3.42, m	70.0, CH^a^	—	—
Rha-4	—	—	3.22–3.42, m	71.8, CH^a^	—	—
Rha-5	—	—	3.22–3.42, m	68.2, CH	—	—
Rha-6	—	—	1.00, d (6.1)	17.7, CH_3_	—	—

^a,b,c,d,e,f^Assignment with the same superscript may be interchanged in the same column.

**Table 6:** ^1^H- and^13^C-NMR spectroscopic data of compounds **8** and **9** in CD_3_OD.

Carbon number	**8**		**9**	
	*δ*_H,_ mult. (*J* in Hz)	*δ*_C_	*δ*_H,_ mult. (*J* in Hz)	*δ*_C_
2	—	164.1, C	—	163.7, C^a^
3	6.20, d (9.5)	112.7, CH	6.18, d (9.5)	114.5, CH^b^
4	7.84, d (9.5)	146.1, CH	7.84, d (9.5)	146.0, CH
5	7.10, s	110.0, CH	7.45, d (8.5)	130.7, CH
6	—	147.1, C	6.77, dd (2.4,8.5)	112.3, CH^b^
7	—	152.9, C^c^	—	163.1, C^a^
8	6.76, s	104.0, CH	6.70, d (2.4)	103.4, CH
9	—	151.5, C^c^	—	157.2, C^c^
10	—	112.6, C	—	111.6, C
OCH_3_	3.90, s	56.8, CH_3_	—	—

^a,b,c^Assignment with the same superscript may be interchanged in the same column.

**Table 7:** ^1^H- and^13^C-NMR spectroscopic data of compound **10** in CD_3_OD.

Carbon number	*δ*_H,_ mult. (*J* in Hz)	*δ*_C_
1	—	136.5, C
2	6.81, d (1.8)	118.3, CH
3	—	146.4, C
4	—	150.8, C
5	7.07, d (8.2)	114.2, CH
6	6.71, dd (8.2, 1.8)	122.1, CH
7	3.31, d (6.7)	40.7, CH_2_
8	5.94, ddt (10.2, 17.0, 6.7)	139.0, CH
9	5.05, dd (17.0, 1.8)	115.1, CH_2_
	5.01, dd (10.2, 1.8)	
OCH_3_	3.83, s	56.8, CH_3_
Glc-1	4.83, d (7.3)	103.1, CH
Glc-2	3.48, dd (7.3, 9.1)	74.9, CH
Glc-3	3.37–3.39, m	78.2, CH^a^
Glc-4	3.45, dd (9.5, 9.1)	71.4, CH
Glc-5	3.37–3.39, m	77.9, CH^a^
Glc-6	3.68, dd (12.2, 5.4)	62.5, CH_2_
	3.86, dd (12.2, 1.5)	

^a^Assignments with the same superscripts may be may be interchanged in the same column.

**Table 8:** ^1^H- and^13^C-NMR spectroscopic data of compound **11** in DMSO-*d*_6_.

Carbon number	*δ*_H,_ mult. (*J* in Hz)	*δ*_C_
1	3.08, dd (4.2, 9.2)	77.9, CH
2	1.36–1.48, m	27.9, CH_2_
3	1.36–1.48, m	39.9, CH_2_
4	—	71.3, C
5	1.74, d (11.6)	49.9, CH
6	4.48, dd (4.6, 11.6)	76.8, CH
7	1.83, m	40.3, CH
8	1.62, d (14.2)	22.4, CH_2_
	1.36–1.48, m	
9	1.36–1.48, m	35.5, CH_2_
	1.14, m	
10	—	41.1, C
11	2.11, dq (7.0, 6.7)	24.6, CH
12	0.90, d (6.7)	22.6, CH_3_
13	1.03, d (6.7)	23.1, CH_3_
14	0.83, s	13.8, CH_3_
15	1.23, s	23.6, CH_3_
Glc-1	4.36, d (7.3)	98.3, CH
Glc-2	2.93, m	74.3, CH
Glc-3	3.10–3.16, m	77.0, CH
Glc-4	3.02–3.04, m	70.4, CH
Glc-5	3.10–3.16, m	76.9, CH
Glc-6	3.42, dd (5.0, 11.2)	61.4, CH_2_
	3.70, dd (4.3, 11.2)	
